# Cost-effectiveness analysis of chromosomal microarray as a primary test for prenatal diagnosis in Hong Kong

**DOI:** 10.1186/s12884-020-2772-y

**Published:** 2020-02-14

**Authors:** Claudia Ching Yan Chung, Kelvin Yuen Kwong Chan, Pui Wah Hui, Patrick Kwok Cheung Au, Wai Keung Tam, Samuel Kai Man Li, Gordon Ka Chun Leung, Jasmine Lee Fong Fung, Marcus Chun Yin Chan, Ho Ming Luk, Annisa Shui Lam Mak, Kwok Yin Leung, Mary Hoi Yin Tang, Brian Hon Yin Chung, Anita Sik Yau Kan

**Affiliations:** 1 Department of Paediatrics and Adolescent Medicine, LKS Faculty of Medicine, The University of Hong Kong, Hong Kong, Special Administrative Region China; 2 Department of Obstetrics and Gynaecology, Queen Mary Hospital, Hong Kong, Special Administrative Region China; 3 Prenatal Diagnostic Laboratory, Tsan Yuk Hospital, Hong Kong, Special Administrative Region China; 4 Department of Health, Clinical Genetic Service, Hong Kong, Special Administrative Region China; 5 Department of Obstetrics and Gynaecology, Queen Elizabeth Hospital, Hong Kong, Special Administrative Region China; 6 Department of Obstetrics and Gynaecology, The University of Hong Kong, Hong Kong, Special Administrative Region China

**Keywords:** Chromosomal microarray - prenatal diagnosis - cost effectiveness analysis - cost saving - Hong Kong, Karyotyping

## Abstract

**Background:**

Chromosomal microarray (CMA) has been shown to be cost-effective over karyotyping in invasive prenatal diagnosis for pregnancies with fetal ultrasound anomalies. Yet, information regarding preceding and subsequent tests must be considered as a whole before the true cost-effectiveness can emerge. Currently in Hong Kong, karyotyping is offered free as the standard prenatal test while genome-wide array comparative genome hybridization (aCGH), a form of CMA, is self-financed. A new algorithm was proposed to use aCGH following quantitative fluorescent polymerase chain reaction (QF-PCR) as primary test instead of karyotyping. This study aims to evaluate the cost-effectiveness of the proposed algorithm versus the current algorithm for prenatal diagnosis in Hong Kong.

**Methods:**

Between November 2014 and February 2016, 129 pregnant women who required invasive prenatal diagnosis at two public hospitals in Hong Kong were prospectively recruited. The proposed algorithm was performed for all participants in this demonstration study. For the cost-effectiveness analysis, cost and outcome (diagnostic rate) data were compared with that of a hypothetical scenario representing the current algorithm. Further analysis was performed to incorporate women’s willingness-to-pay for the aCGH test. Impact of government subsidies on the aCGH test was explored as a sensitivity analysis.

**Results:**

The proposed algorithm dominated the current algorithm for prenatal diagnosis. Both algorithms were equally effective but the proposed algorithm was significantly cheaper (*p* ≤ 0.05). Taking into account women’s willingness-to-pay for an aCGH test, the proposed algorithm was more effective and less costly than the current algorithm. When the government subsidy reaches 100%, the maximum number of diagnoses could be made.

**Conclusion:**

By switching to the proposed algorithm, cost saving can be achieved whilst maximizing the diagnostic rate for invasive prenatal diagnosis. It is recommended to implement aCGH as a primary test following QF-PCR to replace the majority of karyotyping for prenatal diagnosis in Hong Kong.

## Background

Conventional G-banded karyotyping has been the gold standard for chromosomal analysis in prenatal diagnosis for many decades [1–4]. This technology is limited by the resolution of 5–10 Mb to detect chromosomal anomalies and a turn-around time (TAT) of 2 to 3 weeks. This has now been supplemented or replaced by chromosomal microarray (CMA), which is capable of providing high resolution analysis of chromosomal aberrations in a shorter TAT. The effectiveness of its application in prenatal diagnosis over karyotyping has been demonstrated in multiple cohort studies around the world, [5–8] including a study done by our group in Hong Kong [9]. Recent studies recommended wide-spread implementation of CMA as the preferred test for pregnancies with ultrasound anomalies in different parts of the world, [10–15] as well as a valuable diagnostic tool in pregnancy with increased risk at first trimester screening [16]. Potential drawbacks of CMA include its inability to detect balanced chromosomal rearrangements, polyploidy, low level mosaicism and marker chromosomes lacking euchromatic material; though polyploidy and low level mosaicism for common autosomal and sex chromosome aneuploidies can be detected by rapid aneuploidy detection using quantitative fluorescent polymerase chain reaction (QF-PCR) before performing CMA.

Despite compelling evidence on the diagnostic benefits of using CMA for invasive prenatal diagnosis, CMA is not implemented in the Hong Kong public healthcare system. In the current practice in Hong Kong, women who require invasive prenatal diagnosis and are eligible for public healthcare service will be offered free of charge karyotyping. Women with fetal ultrasound abnormality and increased nuchal translucency (NT) will also be offered QF-PCR for rapid aneuploidy detection free of charge. Self-financed CMA is available if the patient is willing to pay for it. The introduction of CMA into routine testing for prenatal diagnosis was mainly hindered by the perception that CMA is significantly more expensive than karyotype. In this demonstration study, we proposed a new algorithm of rapid aneuploidy detection using QF-PCR followed by CMA for all pregnancies undergoing invasive diagnostic procedure. Although CMA was shown to be more cost-effective than karyotyping, [4, 15] the lack of consensus in the combination and sequence of technology choice makes this study important to evaluate the clinical- and cost-effectiveness of incorporating CMA to prenatal diagnosis in the public healthcare system in Hong Kong.

## Methods

### Overall design of the demonstration study (proposed algorithm)

#### Patient and public involvement

Ethics approval was granted by the Institutional Review Board, the University of Hong Kong/Hospital Authority, Hong Kong (IRB reference number UW 14–465) and Research Ethics Committee, Kowloon Central / Kowloon East, Queen Elizabeth Hospital (IRB reference number KC/KE-14-0212/FR-1). Between November 2014 and February 2016, pregnant women who required invasive prenatal diagnosis at Tsan Yuk Hospital and Queen Elizabeth Hospital (both public hospitals under the Hospital Authority) were prospectively recruited. Pretest counseling was given by trained midwives and maternal fetal medicine subspecialists. An information leaflet and a set of diagrams were used to illustrate genome wide array comparative genome hybridization (aCGH), a form of CMA, and karyotyping. Informed written consent was obtained from all women who agreed to participate in the study under the proposed new algorithm. aCGH was performed using PerkinElmer CGX 60 k oligonucleotide array and the cost of it was fully covered by the Prenatal Diagnostic Laboratory, Tsan Yuk Hospital in this study. Primary indications for invasive prenatal diagnostic test include positive Down syndrome (DS) screening result, fetal ultrasound abnormality, and family history of chromosomal abnormality or genetic disorder.

#### Design

**Fig. 1:**
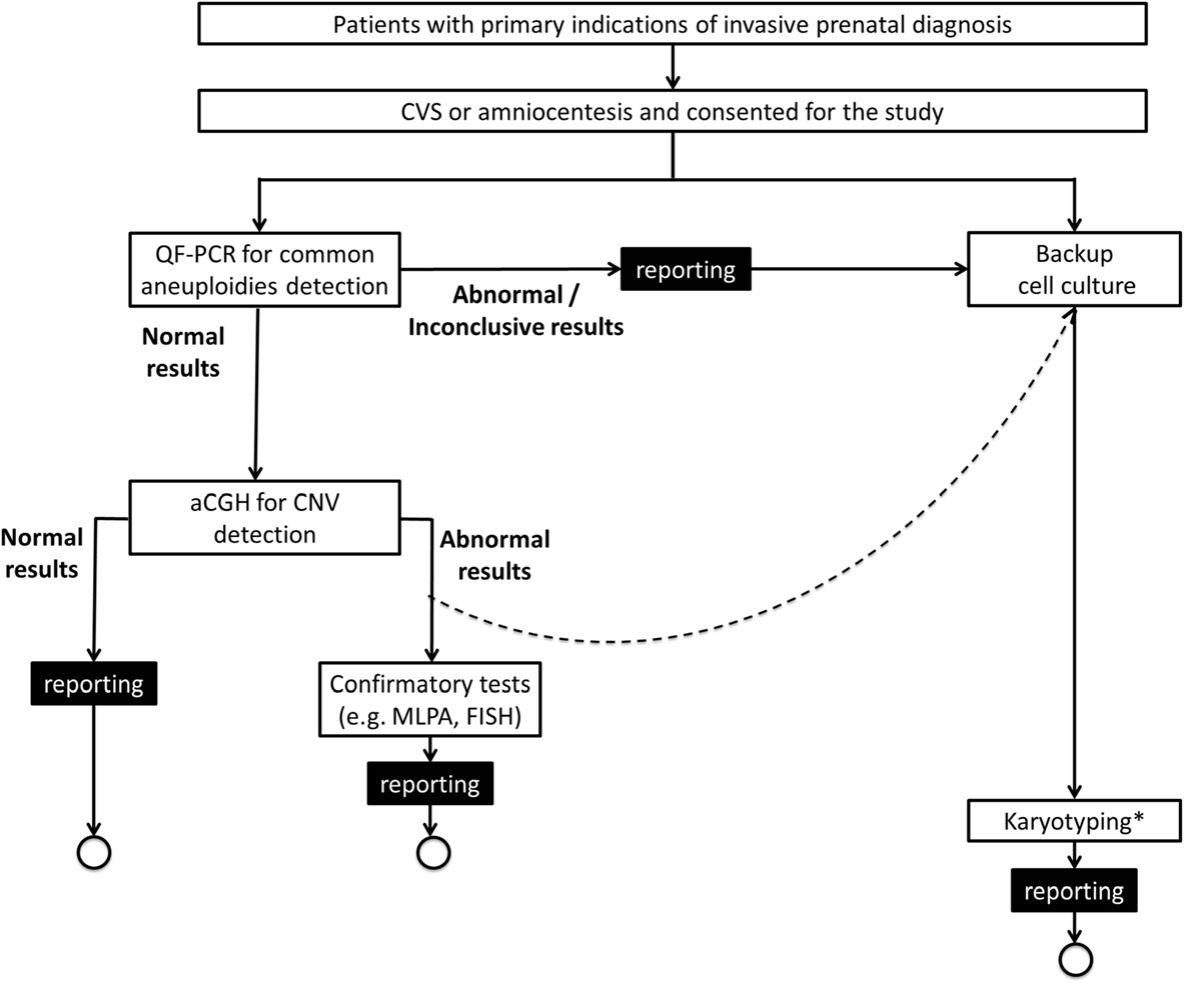
Laboratory workflow of the proposed algorithm for invasive prenatal diagnosis in this study. Rapid aneuploidy detection by QF-PCR will be performed on DNA extracted from the uncultured prenatal samples for all participants who consent for the study, while backup cell culture will also set-up. For those with normal QF-PCR results, they would proceed to aCGH testing. Karyotyping would be performed on backup cell culture for those with abnormal aCGH results (pathogenic or VUS) (indicated by the dotted line arrow), or abnormal (trisomy 13/18/21, monosomy X or triploidy) or inconclusive QF-PCR results. For those with inconclusive QF-PCR results and subsequent normal karyotyping results, aCGH would be performed. If maternal cell contamination could not be excluded by QF-PCR, aCGH would be carried out on cultured cells instead. Laboratory report of the corresponding testing would be issued at each point as indicated in the flowchart. Further confirmatory tests such as fluorescence in-situ hybridization (FISH), multiplex-ligation dependent probe amplification (MLPA), PCR, or parental karyotyping/aCGH, would be considered when aCGH showed abnormal results after discussion with the referring obstetrician. aCGH: array comparative genomic hybridization; CNV: copy number variation; CVS: chorionic villous sampling; FISH: fluorescence in-situ hybridization; MLPA: multiplex-ligation dependent probe amplification; QF-PCR: quantitative fluorescent polymerase chain reaction. *Samples with inconclusive QF-PCR results and subsequent normal karyotyping results will proceed to aCGH on cultured cells

The laboratory workflow of the proposed new algorithm for invasive prenatal diagnosis is illustrated in Fig. 1.

In the proposed algorithm, rapid aneuploidy detection by QF-PCR was performed on DNA extracted from the uncultured prenatal samples for all participants who consented for the study, while backup cell culture was also set-up. For those with normal QF-PCR results, aCGH was performed. Parental CMA was performed to assist interpretation of CMA result of the prenatal sample if necessary. Karyotyping was performed for all abnormal aCGH results (pathogenic or variants of uncertain clinical significance [VUS]), or abnormal (trisomy 13/18/21, monosomy X or triploidy) or inconclusive QF-PCR results. For those with inconclusive QF-PCR results and subsequent normal karyotyping results, aCGH would be performed. Under circumstances where there was maternal cell contamination, aCGH would be performed on cultured cells instead. Further confirmatory tests such as fluorescence in-situ hybridization (FISH), multiplex-ligation dependent probe amplification (MLPA), PCR, or parental karyotyping/aCGH, were performed when aCGH showed abnormal results after discussion with the referring obstetrician.

A laboratory report was released to the referring obstetrician who would provide post-test counseling and follow up for the women. If necessary, referral to clinical geneticists or other subspecialists for assessment and counseling was arranged. Pregnancy outcome was retrieved from the hospital record.

### Economic evaluation

The cost-effectiveness analysis was conducted from the healthcare system perspective. In addition, since CMA remains a self-financed test in Hong Kong, a wider societal perspective was adopted to include patients’ out-of-pocket costs on the prenatal diagnostic tests.

#### Cost estimation

Costs were estimated from the healthcare system perspective and the societal perspective. Healthcare system costs were derived by the summation of the staff costs, reagents and consumable costs, major equipment costs, overhead costs, and other associated costs of each sample. Societal costs included all the healthcare system costs and patients’ out-of-pocket expenditure on the prenatal diagnostic tests. Costs were reported in Hong Kong dollars (HKD) which had an exchange rate of about 7.8 per US dollar at the time of study.

Total costs were calculated for each participant regardless of the outcome. Unit costs obtained were assumed to be a reasonable approximation that reflects the long-run marginal opportunity costs (Table 1). The staff costs was based on unit cost per minute of hands-on-time calculated using the 2017 Hospital Authority staff salary point scale, which included medical consultant, clinical scientist, senior medical technologist, associate medical technologist, and laboratory supporting staff. Unit costs for reagents, consumables, and equipment (including maintenance and service costs) were obtained from price lists provided by laboratory suppliers in 2017. Major equipment cost such as the microarray scanner was calculated based on the equipment predicted lifetime and depreciated using equivalent annual costing. Overhead costs such as electricity, laboratory and building utilities, were calculated as 9–18% of the total costs. Other costs included cell culture and clerical support.

**Table 1:** Cost breakdown of each technology per sample

–	QF-PCR	aCGH /Parental aCGH	AF/CV karyotyping	Blood karyotyping	FISH	MLPA	Other PCR molecular study
	Cost per sample (HKD $)						
Staff	927	2281	1850	1490	1715	1743	687
Reagents and consumables	128	1847	196	196	304	2100	280
Major equipment ^a^	7	169	90	90	137	175	7
Overheads	215	580	314	174	364	918	193
Other costs	23	23	50	50	80	64	33
Total	1300	4900	2500	2000	2600	5000	1200

^a^ Major equipment for QF-PCR includes: DNA fragment analyzer, thermal cycler, and centrifuge. Major equipment for aCGH and parental aCGH includes: microarray scanner, incubator, thermal cycler, spectrophotometer, gel electrophoresis, gel image documentation, and centrifuge. Major equipment for conventional cytogenetics (AF/CV/Blood karyotyping) includes: CytoVision, biosafety cabinets, CO_2_ incubator, and centrifuge. Major equipment for FISH includes fluorescent microscope, thermal hybridizer, and centrifuge. Major equipment for MLPA and other molecular study includes: DNA fragment analyzer, thermal cycler, and centrifuge

*aCGH* array comparative genomic hybridization, *AF/CV* amniotic fluid/chorionic villus, *FISH* fluorescence in-situ hybridization, *MLPA* multiplex-ligation dependent probe amplification, *QF-PCR* quantitative fluorescent polymerase chain reaction

#### Outcome measure

The cost-effectiveness analysis reported here focused on the diagnostic rate (number of diagnoses made/ sample size) as a measure of outcome effectiveness instead of quality adjusted life year (QALY) or life year gained, as the evaluation of QALY and/or life years gained is very challenging in prenatal diagnosis, where the valuation of utilities is limited.

#### Analyses

**Fig. 2:**
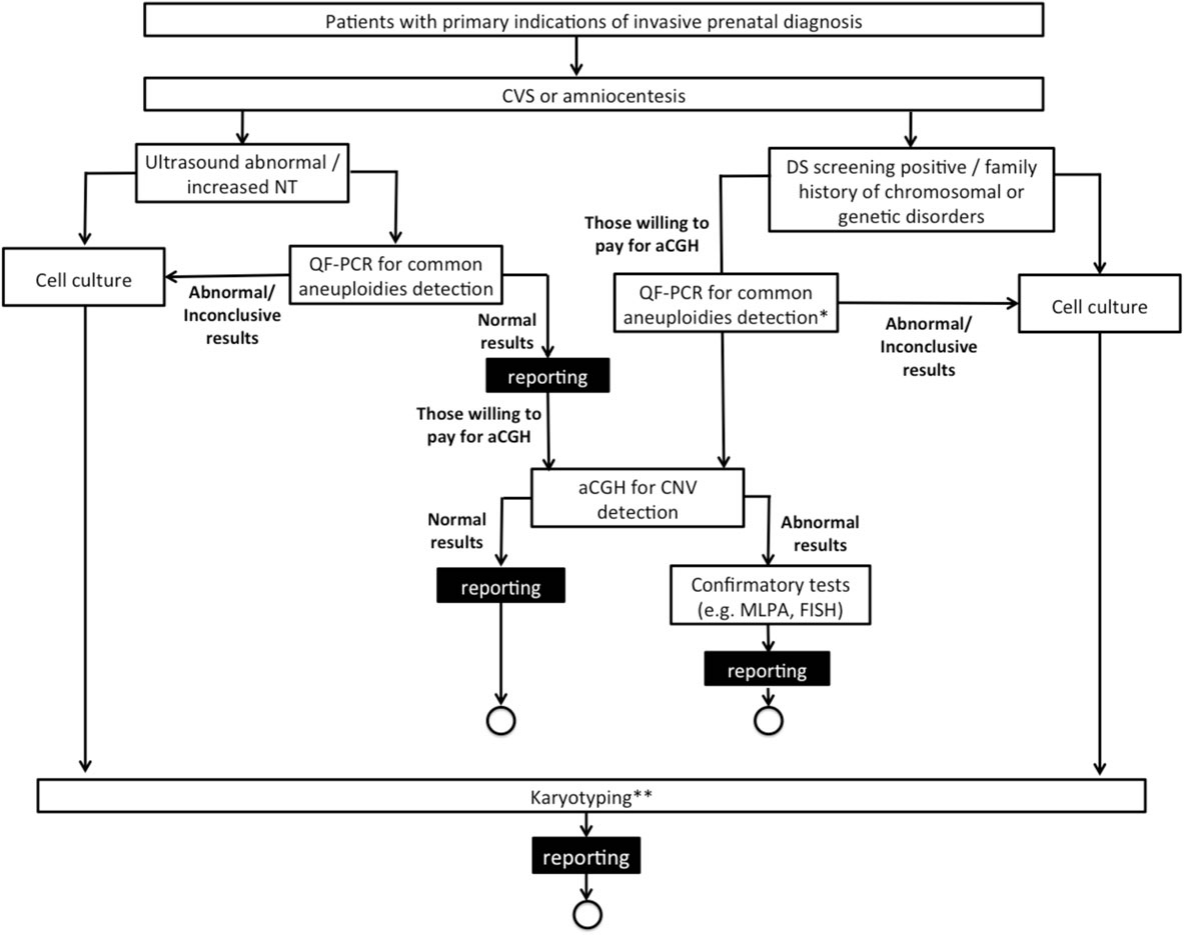
Laboratory workflow of the current algorithm for invasive prenatal diagnosis in the public healthcare system in Hong Kong. *QF-PCR is not commonly offered free of charge for patients with primary indication of DS screening positive / family history of chromosomal or genetic disorders. However, for patients who are willing to pay for self-financed aCGH, the laboratory will first perform QF-PCR for common aneuploidies detection. If QF-PCR results abnormal, aCGH will not be proceeded. ** Samples with inconclusive QFPCR results and subsequent normal karyotyping results will proceed to aCGH if patient is willing to pay for self-financed aCGH. aCGH: array comparative genomic hybridization; CNV: copy number variation; CVS: chorionic villous sampling; DS: Down syndrome; FISH: fluorescence in-situ hybridization; MLPA: multiplex-ligation dependent probe amplification; NT: nuchal translucency; QF-PCR: quantitative fluorescent polymerase chain reaction

To evaluate the cost-effectiveness of the proposed algorithm, the costs and outcomes (diagnostic rate) of this demonstration study were compared with that of a hypothetical scenario which represents the current algorithm of invasive prenatal diagnosis in Hong Kong. The laboratory workflow of the current algorithm for invasive prenatal diagnosis is illustrated in Fig. 2.

In the current algorithm, all patients requiring invasive prenatal testing will be offered amniotic fluid (AF)/chorionic villus (CV) karyotyping. Those with abnormal fetal ultrasound findings and/or increased NT will be offered QF-PCR simultaneously. Self-financed CMA is available to women who are willing to pay $4900. For patients with other primary indications of test such as DS screening positive only, or family history of chromosomal or genetic disorders and are willing to pay for self-financed CMA, the laboratory will also perform QF-PCR for them prior to CMA. The rest of the workflow was similar to the proposed algorithm as described above. Costs and outcome data were estimated by experts and clinicians based on the results from the demonstration study (if the same cohort was to undergo the current algorithm instead of the proposed algorithm).

In the primary analysis, costs and outcomes from the proposed algorithm were compared with that of the current algorithm, under an ideal situation that assumed 100% of the patients are willing to pay 100% out-of-pocket for the aCGH test. In the secondary analysis, unpublished data on willingness-to-pay, which was extracted from the data set collected from the questionnaire used in our previous study [17], on the perceptions of pregnant women and healthcare providers on invasive prenatal testing were incorporated. Only 41.8% of 717 (*n* = 300) women from that study were willing to undergo aCGH with 100% out-of-pocket payment. Therefore, in the secondary analysis, only 41.8% of the patients in this study would be costed for aCGH in the analysis.

Cost data was replicated 1000 times using non-parametric bootstrapping to mitigate the effects of data skewness and to enable quantification of the uncertainty surrounding the estimates of costs and effects by estimating the 95% confidence intervals (CIs). The difference between the two algorithms could be judged to be significant at *p* ≤ 0.05 where the bias-corrected CIs of change scores excluded zero. An incremental cost-effectiveness ratio (ICER) was calculated for each cost-outcome combination that showed higher costs and better outcomes, or lower costs and worse outcomes. This was calculated as the bootstrapped mean cost difference divided by the mean effect (diagnostic rate) difference between the two algorithms. The ICER represents the additional cost for every additional unit of effectiveness (an additional 1% of diagnostic rate) made by the proposed algorithm. Data analyses were conducted using STATA (version 15).

### Sensitivity analysis

A sensitivity analysis was undertaken to assess the impact of uncertainty surrounding the key parameters or methodological features.

As aforementioned, only 41.8% of 717 (*n* = 300) women were willing to undergo aCGH with 100% out-of-pocket payment. In fact, an additional of 53.8% (*n* = 386) was also willing to undergo out-of-pocket aCGH if the cost is less expensive. Therefore, in this sensitivity analysis, the impact of a range of government subsidies on the aCGH test in both of the algorithms was explored. The number of diagnoses made would be based on the diagnostic rate found in this demonstration study. The cost per diagnosis of the proposed algorithm and the current algorithm at each percentage of government subsidies was then compared. The ICER was also explored at each percentage of government subsidies.

## Results

### Primary analysis: assuming 100% of the pregnant women are willing to pay for the self-financed aCGH test

Table 2 compared the outcomes and costs associated with the proposed algorithm and the hypothetical scenario of the current algorithm for invasive prenatal diagnosis in the public healthcare system in Hong Kong. Detailed versions of the proposed and current algorithms with the number of patients following the workflows are illustrated in the Additional file 1: Figure S1a and S1b.

**Table 2:**
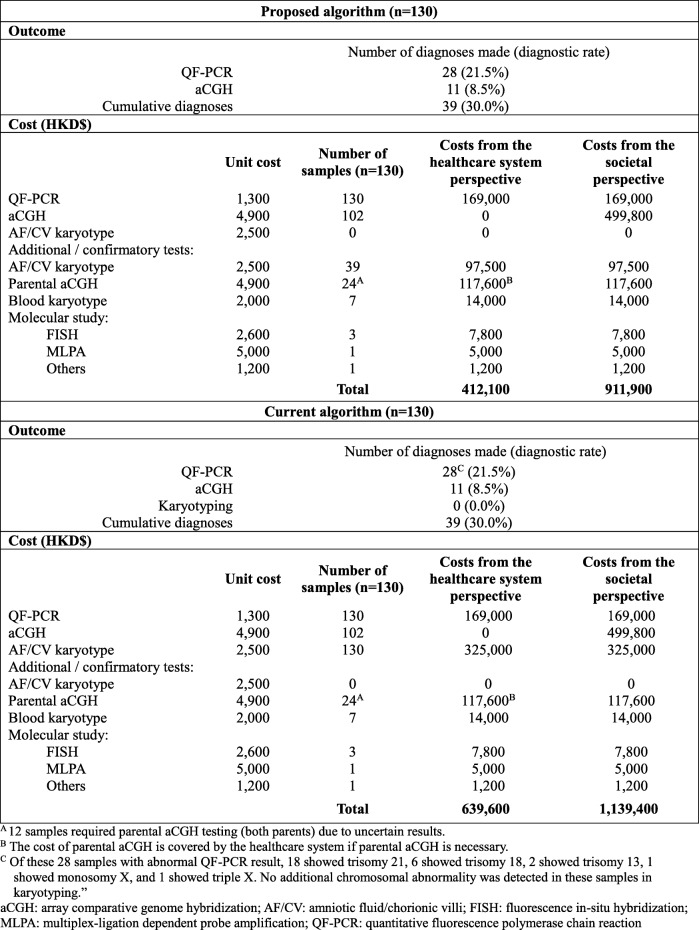
Primary analysis: cost and outcome comparison of the proposed algorithm versus the current algorithm in the public healthcare system in Hong Kong, assuming that 100% of the patients are willing to pay for the out-of-pocket aCGH

#### Demonstration study (proposed algorithm) outcome

From November 2014 to February 2016, 188 women who required invasive prenatal tests with a primary indication for chromosomal study at two obstetric units were recruited to the demonstration study of the proposed algorithm. Only 129 (69.0%) women consented for the study in which one woman was of twin pregnancy, resulting in 130 invasive prenatal diagnostic testing samples. The primary indication for invasive testing of these samples is summarized in Additional file 3: Table S1. Out of the 130 samples, 28 (21.5%) had fetal aneuploidy detected by QF-PCR and therefore did not proceed to aCGH testing (18 had trisomy 21, 6 had trisomy 18, 2 had trisomy 13, 1 had monosomy X, and 1 had triple X). As a result, out of 102 aCGH performed, 11 (10.8%) of them showed abnormal aCGH results (Additional file 3: Table S2). Altogether, the total yield for QF-PCR and aCGH combined was 39 diagnoses (30.0%). AF/CV karyotype was performed for these 39 samples. Twelve prenatal samples required parental aCGH testing (*n* = 24) to investigate inheritance. A total of 7 blood karyotype, 3 FISH, 1 MLPA and 1 PCR were performed as confirmatory/ additional tests after discussing with the clinician. No sample showed an inconclusive QF-PCR result. The pregnancy outcome of the 91 samples with normal aCGH was retrieved from available hospital records, and there were no known missing cases of chromosomal abnormalities detected after birth.

#### Hypothetical scenario (current algorithm) outcome

In order to compare the clinical outcomes between the proposed algorithm and the current algorithm, clinicians and experts estimated the tests that would have been performed for the cohort if they were to undergo the current algorithm instead of the proposed algorithm.

In the primary analysis (assuming 100% of the patients are willing to pay for out-of-pocket aCGH), all samples (*n* = 130) would have undergone AF/CV karyotype. For those with fetal ultrasound abnormality and/or increased NT as the primary indication for invasive testing, QF-PCR would also be performed (*n* = 73) with 20 (15.4%) abnormal results detected within this group. Those with normal QF-PCR results would proceed to self-financed aCGH (*n* = 53). With the additional diagnostic rate of aCGH of 10.8% (11/102 from this demonstration study), aCGH would yield 6 additional diagnoses. Alternatively, for those with positive DS screening results or family history of chromosomal or genetic disorders as primary indications for prenatal diagnosis (*n* = 57), QF-PCR would also be performed prior aCGH as it was assumed that 100% of these patients are willing to pay for the aCGH. In this group, the remaining 8 (6.2%) aneuploidy cases would be detected by QF-PCR. Those with normal QF-PCR results would proceed to self-financed aCGH (*n* = 49), yielding the remaining 5 diagnoses (10.8% of 49). Based on the actual results from the demonstration study (proposed algorithm), the number of parental aCGH tests (*n* = 24) and additional confirmatory tests (7 blood karyotype, 3 FISH, 1 MLPA, and 1 PCR) remained the same. Altogether, a total of 39 diagnoses would be made (30.0%).

AF/CV karyotyping would be able to detect all the 28 diagnoses made by QF-PCR, and 4 out of 11 diagnoses (36.4%) by aCGH (Additional file 3: Table S2, Cases 1–4). TAT of QF-PCR and aCGH are both shorter than karyotyping, which means that karyotyping would not provide the diagnostic result as early as QF-PCR and aCGH under the current algorithm.

#### Incremental costs and outcomes

The incremental costs and outcomes of the proposed algorithm compared with the current algorithm is shown in Table 3.

**Table 3:**
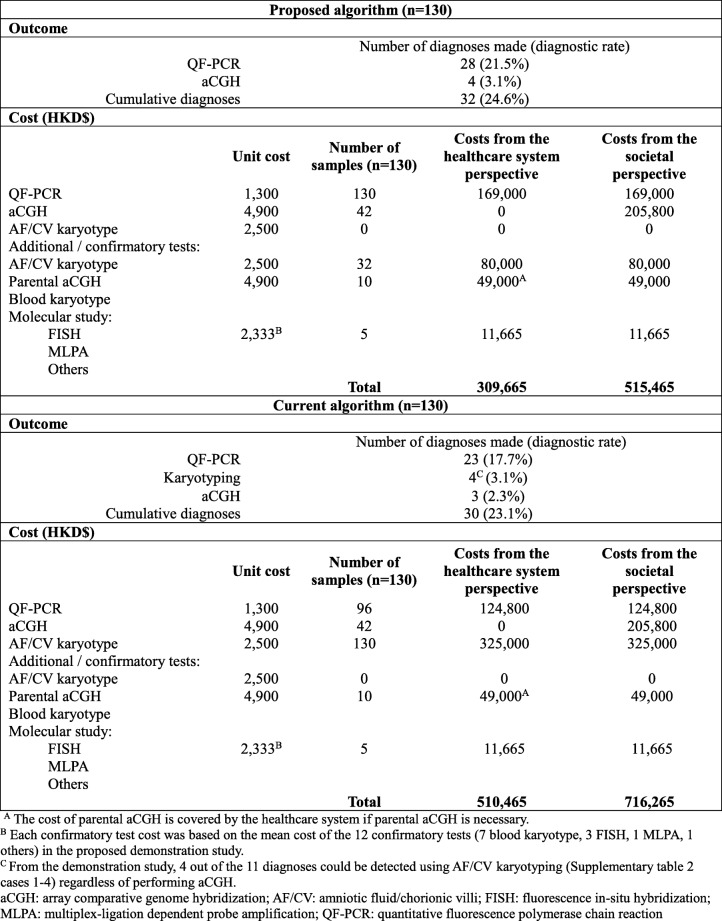
Secondary analysis: cost and outcome comparison of the proposed algorithm versus the current algorithm in the public healthcare system in Hong Kong, with 41.8% of women willing to pay for the out-of-pocket aCGH

In the primary analysis, total costs of the proposed algorithm were lower than that of the current algorithm from both the healthcare system perspective ($412,100 vs. $639,600) and the societal perspective ($911,900 vs. $1,139,400). This was mainly due to the significantly less number of AF/CV karyotype performed in the proposed algorithm. Total cost per sample of the proposed algorithm was significantly cheaper than that of the current algorithm. The proposed algorithm could save $1750 per sample from both the healthcare system perspective (95% CI: -$2395 to -$1098) and from the societal perspective (95% CI: -$2545 to -$817). It could also save $5833 per diagnosis from both perspectives.

Both the proposed and current algorithms yielded the same number of diagnoses (39/130; 30.0%) under the assumption that 100% of the pregnant women requiring aCGH are willing to pay 100% out-pf-pocket for the aCGH test ($4900). The diagnostic rate comparison revealed no significant differences between the algorithms in the primary analysis (0.0, 95% CI: − 12.3 to 10.2%).

#### Cost-effectiveness of the proposed algorithm

**Fig. 3:**
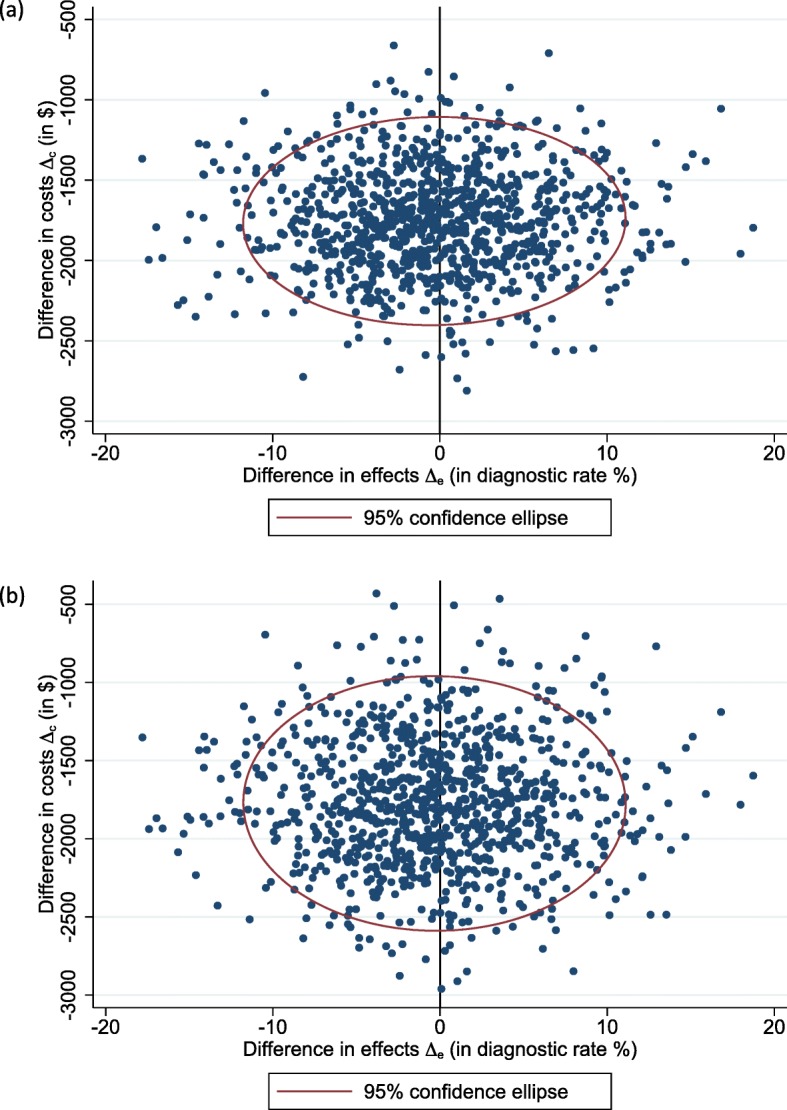
Primary analysis: bootstrapped replicates of incremental cost and incremental diagnostic rate for the proposed algorithm vs. the current algorithm. **a** Healthcare system perspective; and **b** societal perspective

In the primary analysis, under both perspectives, the proposed algorithm was dominant (Table 3). Figure 3 shows 1000 bootstrapped replicates of incremental costs and incremental diagnostic rate from both perspectives. All the 1000 bootstrapped resamples involved cost-savings in the proposed algorithm compared with the current algorithm from both the healthcare system (95% C.I.: -$2395 to -$1098) and societal perspectives (95% C.I.: -$2545 to -$817).

### Secondary analysis: assuming only 41.8% of pregnant women requiring aCGH are willing to pay for the self-financed aCGH test

Table 3 compared the costs and outcome associated with the proposed algorithm and the hypothetical scenario of the current algorithm for invasive prenatal diagnosis in the public healthcare system in Hong Kong. In this secondary analysis, the proposed algorithm assumed that 41.8% of women undergoing invasive prenatal diagnosis are willing to pay for self-financed aCGH test. Those who are not willing to pay for aCGH would receive prenatal diagnosis results based on QF-PCR only and no karyotyping would be performed. Detailed versions of the proposed and current algorithms with the number of patients following the workflows are illustrated in the Additional file 2: Figures S2a and S2b.

#### Hypothetical scenario (proposed algorithm) outcome

For the secondary analysis, all samples would have underwent QF-PCR (*n* = 130) and 28 abnormal QF-PCR results would be picked up (21.5%). Only 41.8% of the women would pay out-of-pocket for an aCGH test as mentioned above, giving a total of 42 aCGH tests being performed (41.8% of 102). Based on the results from the demonstration study (primary analysis), 10.8% of those who undergone aCGH would have abnormal results and 4 diagnoses would be detected (3.1%). A total of 32 AF/CV karyotyping would be performed. It was estimated that 10 parental aCGH (41.8% of the 12 prenatal samples who required parental aCGH from the demonstration study) and 5 additional confirmatory tests (11.8% of aCGH samples) would be needed. The number of confirmatory tests needed (*n* = 5) was based on the percentage found in the demonstration study (11.8% of 102 samples [7 blood karyotype and 5 molecular studies]). The proposed algorithm could yield a total of 32 diagnoses (24.6%) when the proportion of patients willing to pay for self-financed aCGH was incorporated.

#### Hypothetical scenario (current algorithm) outcome

Similar to the primary analysis, QF-PCR would be performed for all patients with the primary indications for invasive testing due to fetal ultrasound abnormality and/or increased NT (*n* = 73), this would pick up 20 (15.4%) aneuploidy cases. For those with positive DS screening results and/or family history of chromosomal or genetic disorders as the primary indications for prenatal diagnosis (*n* = 57), only those who are willing to pay for the self-financed aCGH would be offered QF-PCR (41.8% of 57; *n* = 23). By projecting the results from the demonstration study that the diagnostic rate for QF-PCR in this group of patients was 14.0% (8/57), 3 additional diagnoses could be made in this scenario (14.0% of 23). Therefore, a total of 23 diagnoses could be made by QF-PCR.

All invasive prenatal samples would be offered AF/CV karyotyping under the current algorithm (*n* = 130). In addition to QF-PCR, karyotyping could pick up 4 diagnoses based on the results from the demonstration study (4 out of 11 diagnoses made by aCGH could be detected using AF/CV karyotyping regardless of performing aCGH). Those with normal QF-PCR results and are willing to pay for the self-financed aCGH (*n* = 22 + 20) would continue to proceed to aCGH. With the additional diagnostic rate of aCGH of 7.1% (7/98), aCGH would yield 3 extra diagnoses. It was estimated that 10 parental aCGH (41.8% of the 12 samples who required parental aCGH from the demonstration study) and 5 additional confirmatory tests (11.8% of aCGH samples) would be needed. As a result, a total of 30 diagnoses (23.1%) could be detected.

#### Incremental costs and outcomes

The incremental costs and outcomes of the proposed algorithm compared with the current algorithm is shown in Table 4.

**Table 4:** Incremental costs ($, 2017 prices) and outcomes (diagnostic rate), and incremental cost-effectiveness ratios for the proposed algorithm versus the current algorithm

	Proposed algorithm (*n* = 130)	Current algorithm (*n* = 130)	Difference
**Primary analysis: assuming 100% of the patients are willing to pay for out-of-pocket aCGH**			
**Outcome:**			
Number of diagnosis	39	39	0
Diagnostic rate (%)	30.0	30.0	0.0 (−12.3 to 10.2)
**Total healthcare costs ($)**			
Total costs	412,100	639,600	− 227,500
Total cost per sample	3170	4920	− 1750 (95% CI: − 2395 to − 1098)
Total cost per diagnosis ($/dx)	10,567	16,400	−5833
**Total societal costs ($)**			
Total costs	911,900	1,139,400	−227,500
Total cost per sample	7015	8765	−1750 (95% CI: −2545 to −817)
Total cost per diagnosis ($/dx)	23,382	29,215	−5833
**Healthcare system perspective: Cost per one additional diagnosis (ICER)**			**Proposed algorithm dominates**
**Societal perspective: Cost per one additional diagnosis (ICER)**			**Proposed algorithm dominates**
**Secondary analysis: only 41.8% of the patients are willing to pay for out-of-pocket aCGH**			
**Outcome:**			
Number of diagnosis	32	30	0
Diagnostic rate (%)	24.6	23.1	1.5 (−10.4 to 11.7)
**Total healthcare costs ($)**			
Total costs	309,665	510,465	−200,800
Total cost per sample	2382	3927	−1545 (95% CI:-2030 to −1095)
Total cost per diagnosis ($/dx)	9677	18,231	−7339
**Total societal costs ($)**			
Total costs	515,465	716,265	−200,800
Total cost per sample	3965	5510	−1545 (95% CI: −2407 to −706)
Total cost per diagnosis ($/dx)	16,108	23,876	−7768
**Healthcare system perspective: Cost per one additional diagnosis (ICER)**			**Proposed algorithm dominates**
**Societal perspective: Cost per one additional diagnosis (ICER)**			**Proposed algorithm dominates**

*aCGH* array comparative genome hybridization, *CI* confidence interval, *dx* diagnosis, *ICER* incremental cost-effectiveness ratio

In the secondary analysis, total costs of the proposed algorithm were lower than that of the current algorithm from both the healthcare system perspective ($309,665 vs. $510,465) and the societal perspective ($515,465 vs. $716,265). Total cost per sample of the proposed algorithm was significantly lower than that of the current algorithm. The proposed algorithm could save $1545 per sample from both the healthcare system perspective (95% CI: -$2030 to -$1095) and from the societal perspective (95% CI: -$2407 to -$706). It could also save $7339 per diagnosis from the healthcare system perspective and $7768 per diagnosis from the societal perspective.

Under the assumption that only 41.8% of the pregnant women are willing to pay for out-of-pocket aCGH, the proposed algorithm could yield 2 additional diagnoses than the current algorithm. The diagnostic rate comparison revealed no significant differences between the algorithms (− 1.5, 95% CI: − 10.4 to 11.7%).

#### Cost-effectiveness of the proposed algorithm

**Fig. 4:**
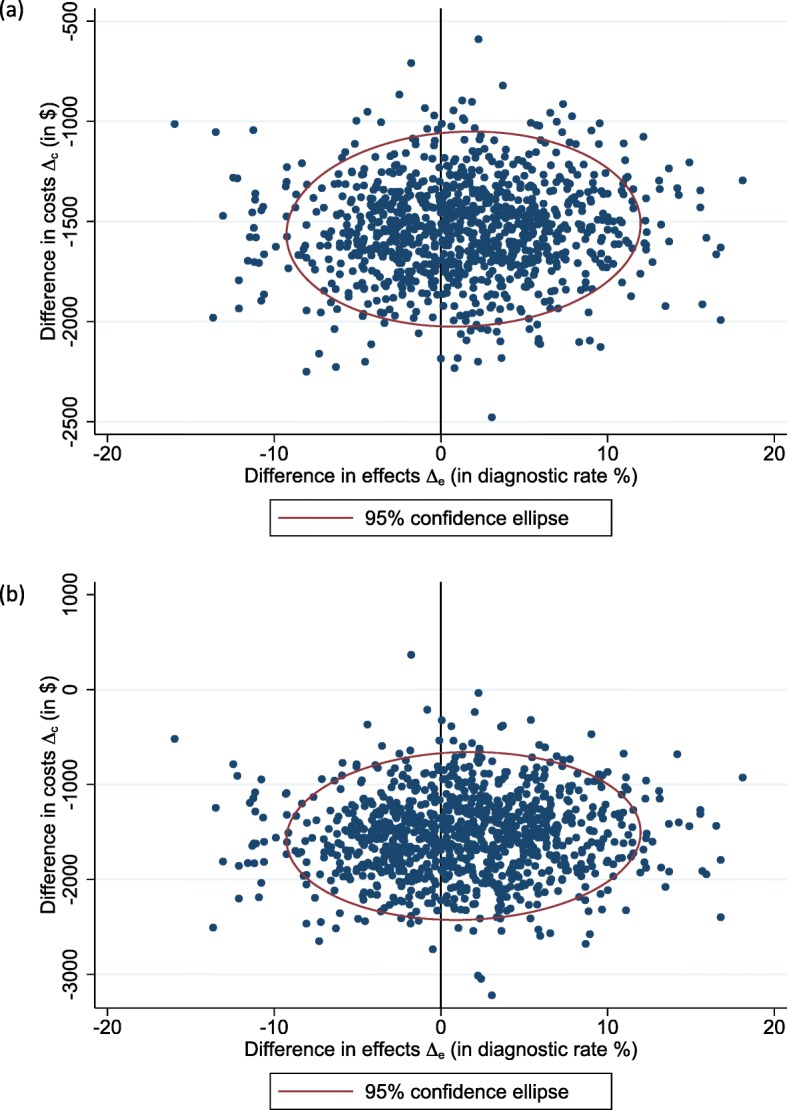
Secondary analysis: bootstrapped replicates of incremental cost and incremental diagnostic rate for the proposed algorithm vs. the current algorithm. **a** Healthcare system perspective; and **b** societal perspective

In the secondary analysis, under both perspectives, the proposed algorithm was dominant (Table 4). Figure 4 shows 1000 bootstrapped replicates of incremental costs and incremental diagnostic rate from both perspectives. From the healthcare system perspective, all the 1000 bootstrapped resamples involved cost-savings in the proposed algorithm compared with the current algorithm (95% C.I.: -$2030 to − 1095); whereas from the societal perspective, over 95% of the bootstrapped resamples involved cost-savings.

### Sensitivity analysis

**Fig. 5:**
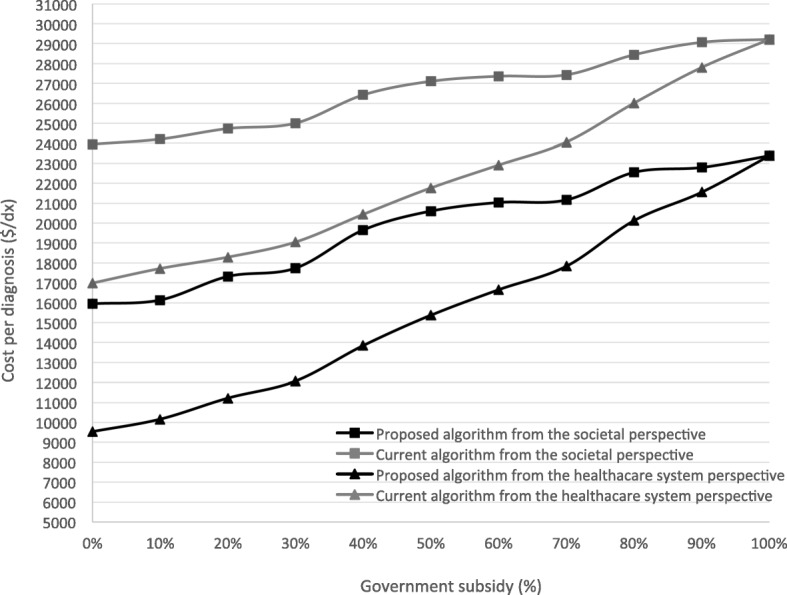
Sensitivity analysis: cost per diagnosis ($/dx) of the proposed algorithm versus the current algorithm based on the percentage of government subsidies

The sensitivity analysis explored the impact of government subsidy on the aCGH test in both of the algorithms (0–100%), at 10% increment (Fig. 5). From both the healthcare system and societal perspectives, the total costs of the proposed algorithm was significantly lower than that of the current algorithm at any percentage of government subsidies (0–100%). In addition, as the government subsidy on the aCGH test increased, the diagnostic rate of both algorithms increased because more patients are willing to pay for the aCGH test at a lower cost. The diagnostic rate of the proposed algorithm was higher than that of the current algorithm at any percentage of government subsidies. Both of the algorithms could reach the maximum number of diagnoses (*n* = 39) when the government subsidy on the aCGH test reaches 100%. The cost per diagnosis of the proposed algorithm from the societal perspective was even cheaper than that of the current algorithm from the healthcare system perspective, at any given point of government subsidy. As a result, it was found that the proposed algorithm dominates the current algorithm for invasive prenatal diagnosis at any point of government subsidy on the aCGH test.

## Discussion

This economic evaluation assessed the cost-effectiveness of the proposed algorithm over the current algorithm for invasive prenatal diagnosis in the public healthcare system in Hong Kong.

The prospective demonstration study reported the successful implementation of aCGH incorporating karyotyping after QF-PCR for prenatal diagnosis in two obstetric units in Hong Kong, which accounted for around nearly 10,000 deliveries and over 10,000 antenatal appointments per year (average of year 2016 and 2017). CMA undoubtedly offers the greatest diagnostic capability, as shown in this demonstration study and in other previous studies [3–7]. The additional diagnostic rate of aCGH was 10.8% (11/102) following rapid aneuploidy by QF-PCR, while the additional diagnostic rate of karyotyping following QF-PCR was only 3.9% (4/102); diagnoses made by karyotyping could all be achieved by aCGH. The 7.1% (7/98) increased diagnostic yield of aCGH in the presence of normal karyotype is consistent with findings from reported literature [8].

From a single test perspective, an aCGH test nearly doubled the unit cost of karyotyping, which in part, explained the hesitation to fund aCGH in routine prenatal diagnostic testing. Yet, the situation is often more complex in reality because information regarding preceding and subsequent tests must also be considered as a whole before the true cost-effectiveness can emerge. It was found that the proposed algorithm (demonstration study) was significantly cheaper than the current algorithm for invasive prenatal diagnosis in Hong Kong. From the primary and secondary analyses, it could save money from both the healthcare system and societal perspectives simply by switching from the current algorithm to the proposed algorithm. In the ideal situation where all women requiring aCGH are willing to pay 100% out-of-pocket for the aCGH test, the current algorithm could only best perform like the proposed algorithm (equally effective in terms of diagnostic rate), but it was significantly more expensive. Thus the proposed algorithm dominated the current practice for invasive prenatal diagnosis in the public healthcare system in Hong Kong. With approximately 1400 invasive prenatal diagnosis tests performed per year, switching to the proposed algorithm could save over $2,000,000 annually. When the patients’ willingness-to-pay on the aCGH test was considered, the diagnostic rate was suboptimal but still comparatively better than that of the current algorithm. When only 41.8% of patients were willing to pay for the out-of-pocket aCGH at full price (*n* = 42), a total of 32 diagnoses and 30 diagnoses could be made under the proposed and current algorithm, respectively. This is at sacrifice of missing 17.9% (7/39) diagnoses under the proposed algorithm, and missing 23.1% (9/39) diagnoses under the current algorithm, which can be overcome by offering aCGH to every patient requiring it.

A similar study in the United Kingdom (UK) by Robson et al. in 2017 [15] evaluated the cost-effectiveness of CMA replacing karyotyping in the prenatal diagnosis pathway of fetal anomalies and found that the ICER was £4703. By evaluating the whole workflow, they have concluded that CMA is a robust and probably cost-effective method to detect more diagnoses and suggested to replace karyotyping with CMA. Our study further strengthened their conclusion, showing a clear dominance of using CMA to replace majority of karyotyping for prenatal diagnosis, though the algorithms in Hong Kong and in the UK were slightly different.

In reality, the diagnostic yield would be compromised without government subsidy. The sensitivity analysis illustrated that the proposed algorithm dominates the current algorithm at any percentage of government subsidies. Yet, it should be highlighted that the maximum diagnostic rate could only be achieved when the government subsidy on the aCGH test reaches 100%.

The introduction of aCGH into routine testing to replace most of the karyotyping for prenatal diagnosis does not only improve diagnostic yield and reduce healthcare system and societal costs, it also allows shortened TAT of prenatal diagnosis. In order to estimate the improvement of TAT of prenatal diagnosis by implementing the proposed algorithm, TAT of aCGH testing (counting from the date of aCGH set-up to reporting) of the 102 cases requiring aCGH in this demonstration study was compared with the TAT of cytogenetic analysis of prenatal samples that were not recruited in the study in the same study period (*n* = 348). There was an overall 5 days of shorter reporting time for 77% of the recruited samples with normal QF-PCR result (*p* < 0.05, Mann Whitney U test), and the difference was up to 8 days when calculated from sample setup to reporting. The shortened TAT highlighted the patient benefits of the proposed algorithm. With shorter waiting time, it decreased the anxiety for couples awaiting test results.

### Strengths and limitations

This study proposed a new algorithm for invasive prenatal diagnosis and fills an important evidence gap, in which it provides the first available evidence of the cost-effectiveness of the algorithms in prenatal diagnosis in the public healthcare system in Hong Kong. This study also explored both the healthcare system costs and impacts on patients’ out-of-pocket aCGH cost as part of a wider societal perspective.

The economic impact of pregnancy continuation or termination and its associated cost for long term follow up was not considered in this analysis. It can be argued that there is potentially significant issue with regard to healthcare system costs due to the additional cases identified and thus the additional pregnancy terminations. Though would be difficult to estimate, it can be imagined that the societal cost will be increased substantially for pregnancy continuation, leading to the same conclusion that the proposed algorithm dominates the current algorithm. This may include potential direct healthcare costs and indirect costs such as loss of productivity of the individual, his/her family and carers, and the society as a whole. In addition, post-test counseling cost was not included in this study; however, it is unlikely that there would be major differences between the two algorithms, as shown by the equal effectiveness in the primary analysis. Although the sample size presented here was relatively small, this analysis presented the bootstrapped point estimates with 95% CIs which should have mitigated the effect of data skewness.

The inability to detect balanced chromosomal rearrangements (BCRs) is a known limitation of CMA. There is a chance that BCRs may be missed using the proposed algorithm. A recent study by Halgren et al. (2018) suggested that cases with de novo BCRs are associated with a higher morbidity risk of 27% developing neurodevelopmental and/or neuropsychiatric disorders than a matched control [18]. Since this is a prospective study that evaluates the diagnostic capacity and cost-effectiveness of the proposed algorithm, long-term follow-up data of the 91 cases with normal aCGH results was not available at the time of conducting the study. Currently, the proposed algorithm with the implementation of aCGH as a primary test is already the better option in terms of costs and diagnostic yield in prenatal cases with structural anomalies as compared to the current algorithm. The feasibility of using mate-pair whole genome sequencing approach to detect BCRs is demonstrated in emerging studies. Nevertheless, not until the cost of this approach falls to a more affordable price and that its cost-effectiveness has been proven as a standard routine test in the public healthcare system, CMA should still be used as a primary invasive prenatal diagnostic test following rapid aneuploidy detection.

Another limitation of the study was the use of a simple outcome measure, diagnostic rate, rather than a health-related outcome such as QALYs. However, the use of QALYs in the prenatal population is not appropriate as the resulting conditions are heterogeneous; the valuation of utilities is limited with the only option being pregnancy continuation or termination. Furthermore, the cost-effectiveness analysis was not based on a randomized controlled study due to budget constraint. The cost-effectiveness analysis compared the prospective demonstration study of the proposed algorithm with a hypothetical scenario, though the scenario represents the current algorithm for invasive prenatal diagnosis in Hong Kong based on actual data collected from the demonstration study. Lastly, the intangible benefits were not presented in this cost-effectiveness analysis, such as informing prenatal and postnatal management decisions, estimating recurrence risk, facilitating delivery and future reproductive plans, etc., which are invaluable and important for patients and healthcare providers. As a result, the cost-effectiveness of the implementation of the proposed algorithm is likely to be underestimated in this study.

Furthermore, non-invasive prenatal test (NIPT) for trisomy 13, 18, 21, as a contingent test following positive DS screening test result would be implemented in the public healthcare system in 2019. This would lead to reduction in invasive prenatal testing for those who had false positive DS screening test result due to conventional screening method. The cost for diagnosis by implementing CMA as primary test thus is anticipated to be even lowered.

## Conclusion

Despite its limitations, the current study provides important evidence that the proposed algorithm is cost saving whilst maximizing the number of diagnoses achieved for invasive prenatal diagnosis in the public healthcare system in Hong Kong. Technology advancement involving next generation sequencing and software improvements such as automation are likely to further increase diagnostic rate, reduce costs, and shorten TAT. It is therefore recommended to switch to the proposed algorithm, with the implementation of aCGH as a routine test for invasive prenatal diagnosis following QF-PCR, to facilitate the uptake of such advances into the Hong Kong public healthcare system through evidence of clinical- and cost-effectiveness. Future areas for research should include establishing the willingness-to-pay thresholds in the local setting to guide decision makers for efficient allocation of healthcare resources.

## Supplementary information

**Additional file 1: Figure S1a** Primary analysis: detailed workflow of the proposed algorithm. Rapid aneuploidy detection by QF-PCR will be performed on DNA extracted from the uncultured prenatal samples for all participants who consent for the study, while backup cell culture will also set-up. For those with normal QF-PCR results, they would proceed to aCGH testing. Karyotyping would be performed on backup cell culture for those with abnormal aCGH results (pathogenic or VUS) (indicated by the dotted line arrow), or abnormal (trisomy 13/18/21, monosomy X or triploidy) or inconclusive QF-PCR results. For those with inconclusive QF-PCR results and subsequent normal karyotyping results, aCGH would be performed. If maternal cell contamination could not be excluded by QF-PCR, aCGH would be carried out on cultured cells instead. Laboratory report of the corresponding testing would be issued at each point as indicated in the flowchart. Further confirmatory tests such as fluorescence in-situ hybridization (FISH), multiplex-ligation dependent probe amplification (MLPA), PCR, or parental karyotyping/aCGH, would be considered when aCGH showed abnormal results after discussion with the referring obstetrician. aCGH: array comparative genomic hybridization; CNV: copy number variation; CVS: chorionic villous sampling; FISH: fluorescence in-situ hybridization; MLPA: multiplex-ligation dependent probe amplification; QF-PCR: quantitative fluorescent polymerase chain reaction. *Samples with inconclusive QF-PCR results and subsequent normal karyotyping results will proceed to aCGH on cultured cells. **Figure S1b** Primary analysis: detailed workflow of the current algorithm. *QF-PCR is not commonly offered free of charge for patients with primary indication of DS screening positive / family history of chromosomal or genetic disorders. However, for patients who are willing to pay for self-financed aCGH, the laboratory will first perform QF-PCR for common aneuploidies detection. If QF-PCR results abnormal, aCGH will not be proceeded. ** Samples with inconclusive QFPCR results and subsequent normal karyotyping results will proceed to aCGH if patient is willing to pay for self-financed aCGH. aCGH: array comparative genomic hybridization; CNV: copy number variation; CVS: chorionic villous sampling; DS: Down syndrome; FISH: fluorescence in-situ hybridization; MLPA: multiplex-ligation dependent probe amplification; NT: nuchal translucency; QF-PCR: quantitative fluorescent polymerase chain reaction.

**Additional file 2: Figure S2a** Secondary analysis: detailed workflow of the proposed algorithm. *Samples with inconclusive QF-PCR results and subsequent normal karyotyping results will proceed to aCGH on cultured cells. aCGH: array comparative genomic hybridization; CNV: copy number variation; CVS: chorionic villous sampling; FISH: fluorescence in-situ hybridization; MLPA: multiplex-ligation dependent probe amplification; QF-PCR: quantitative fluorescent polymerase chain reaction. **Figure S2b** Secondary analysis: detailed workflow of the current algorithm. *QF-PCR is not commonly offered free of charge for patients with primary indication of DS screening positive / family history of chromosomal or genetic disorders. However, for patients who are willing to pay for self-financed aCGH, the laboratory will first perform QF-PCR for common aneuploidies detection. If QF-PCR results abnormal, aCGH will not be proceeded.** Samples with inconclusive QFPCR results and subsequent normal karyotyping results will proceed to aCGH if patient is willing to pay for self-financed aCGH. aCGH: array comparative genomic hybridization; CNV: copy number variation; CVS: chorionic villous sampling; DS: Down syndrome; FISH: fluorescence in-situ hybridization; MLPA: multiplex-ligation dependent probe amplification; NT: nuchal translucency; QF-PCR: quantitative fluorescent polymerase chain reaction.

**Additional file 3: Table S1** Primary indication of invasive testing for 130 prenatal cases. **Table S2** Abnormal aCGH results and outcome (*n* = 11).

## Data Availability

The datasets used and/or analysed during the current study are available from the corresponding authors on reasonable request.

## Abbreviations

aCGH: Array comparative genome hybridization
AF/CV: Amniotic fluid/chorionic villus
BCRs: Balanced chromosomal rearrangements
CIs: Confidence intervals
CMA: Chromosomal microarray
CNV: Copy number variation
CVS: Chorionic villous sampling
DS: Down syndrome
FISH: Fluorescence in-situ hybridization
HKD: Hong Kong dollars
ICER: Incremental cost-effectiveness ratio
MLPA: Multiplex-ligation dependent probe amplification
NIPT: Non-invasive prenatal test
NT: Nuchal translucency
QALY: Quality adjusted life year
QF-PCR: Quantitative fluorescent polymerase chain reaction
TAT: Turn-around time
UK: United Kingdom
VUS: Variants of uncertain clinical significance
