# Supplementary material for: Cost-effectiveness analysis of chromosomal microarray as a primary test for prenatal diagnosis in Hong Kong
Source: BMC Pregnancy Childbirth. 2020 Feb 14;20:109. doi: 10.1186/s12884-020-2772-y (PMC7023733; doi:10.1186/s12884-020-2772-y)

Supplementary figure 1a. Primary analysis: detailed workflow of the proposed algorithm

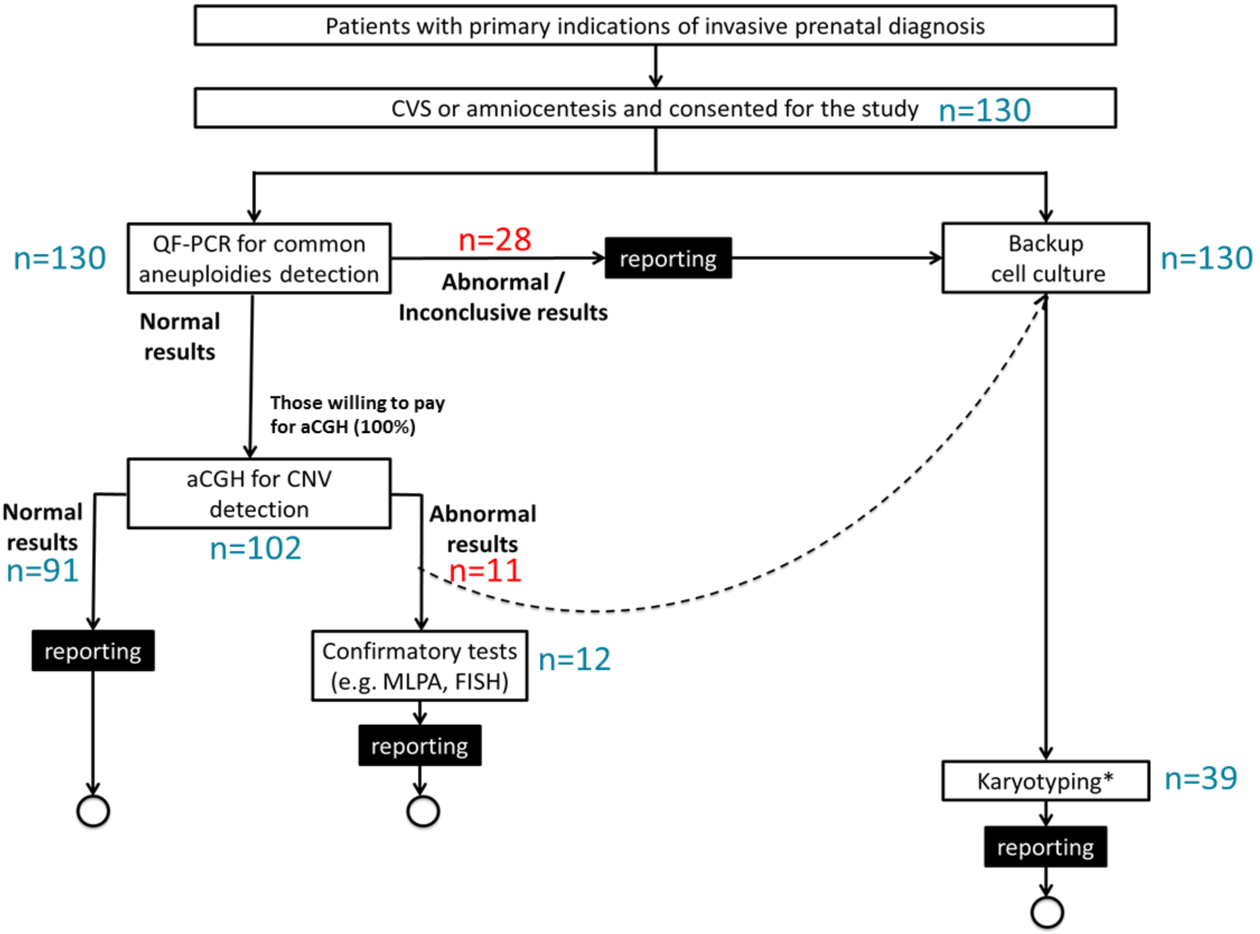

Supplementary figure 1b. Primary analysis: detailed workflow of the current algorithm

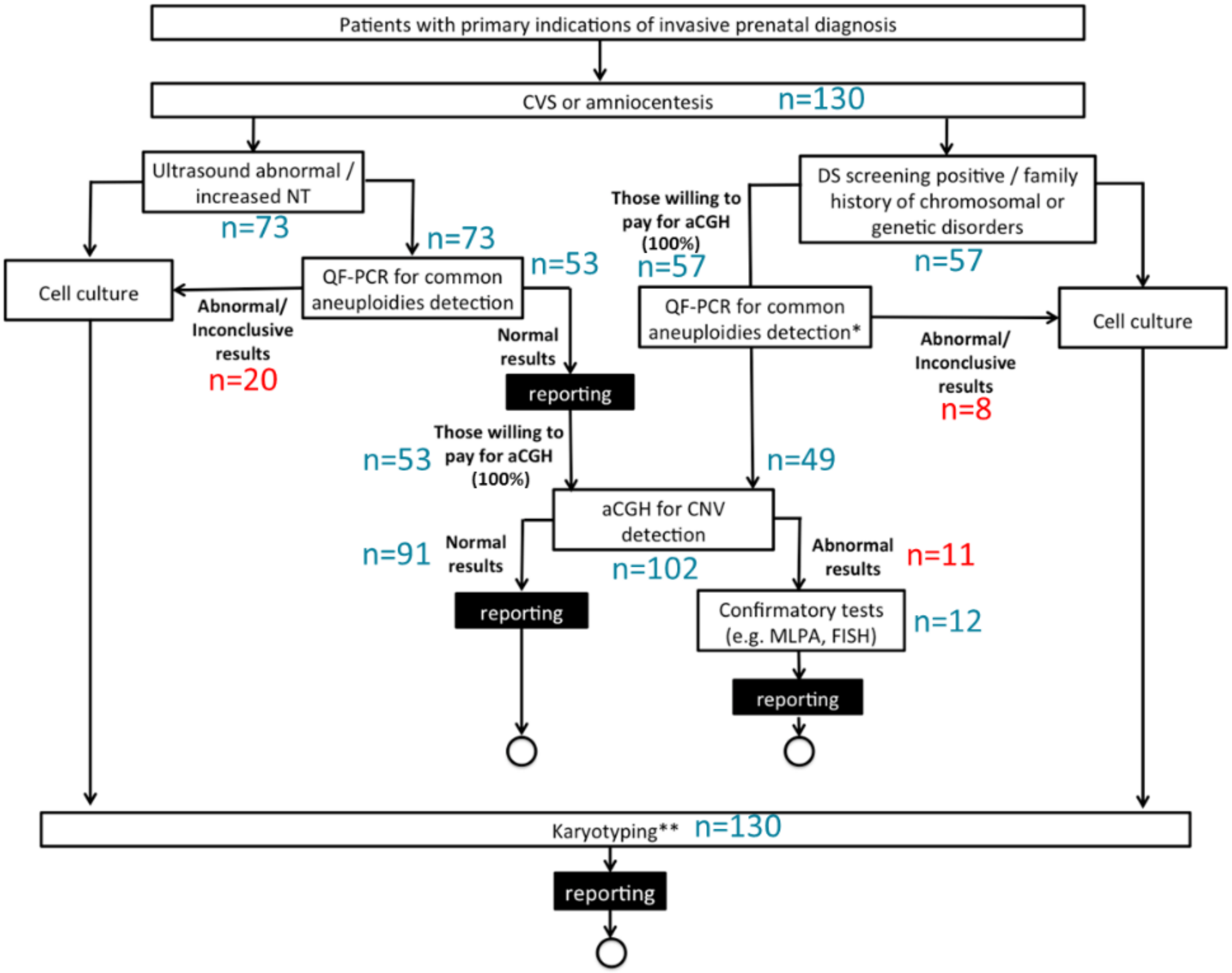

Supplement: Supplementary file 1 — Additional file 1: Figure S1a Primary analysis: detailed workflow of the proposed algorithm. Rapid aneuploidy detection by QF-PCR will be performed on DNA extracted from the uncultured prenatal samples for all participants who consent for the study, while backup cell culture will also set-up. For those with normal QF-PCR results, they would proceed to aCGH testing. Karyotyping would be performed on backup cell culture for those with abnormal aCGH results (pathogenic or VUS) (indicated by the dotted line arrow), or abnormal (trisomy 13/18/21, monosomy X or triploidy) or inconclusive QF-PCR results. For those with inconclusive QF-PCR results and subsequent normal karyotyping results, aCGH would be performed. If maternal cell contamination could not be excluded by QF-PCR, aCGH would be carried out on cultured cells instead. Laboratory report of the corresponding testing would be issued at each point as indicated in the flowchart. Further confirmatory tests such as fluorescence in-situ hybridization (FISH), multiplex-ligation dependent probe amplification (MLPA), PCR, or parental karyotyping/aCGH, would be considered when aCGH showed abnormal results after discussion with the referring obstetrician. aCGH: array comparative genomic hybridization; CNV: copy number variation; CVS: chorionic villous sampling; FISH: fluorescence in-situ hybridization; MLPA: multiplex-ligation dependent probe amplification; QF-PCR: quantitative fluorescent polymerase chain reaction. *Samples with inconclusive QF-PCR results and subsequent normal karyotyping results will proceed to aCGH on cultured cells. Figure S1b Primary analysis: detailed workflow of the current algorithm. *QF-PCR is not commonly offered free of charge for patients with primary indication of DS screening positive / family history of chromosomal or genetic disorders. However, for patients who are willing to pay for self-financed aCGH, the laboratory will first perform QF-PCR for common aneuploidies detection. If QF-PCR resu [file 12884_2020_2772_MOESM1_ESM.pdf]
