# Supplementary material for: Cost-effectiveness analysis of chromosomal microarray as a primary test for prenatal diagnosis in Hong Kong
Source: BMC Pregnancy Childbirth. 2020 Feb 14;20:109. doi: 10.1186/s12884-020-2772-y (PMC7023733; doi:10.1186/s12884-020-2772-y)

Supplementary figure 2a. Secondary analysis: detailed workflow of the proposed algorithm

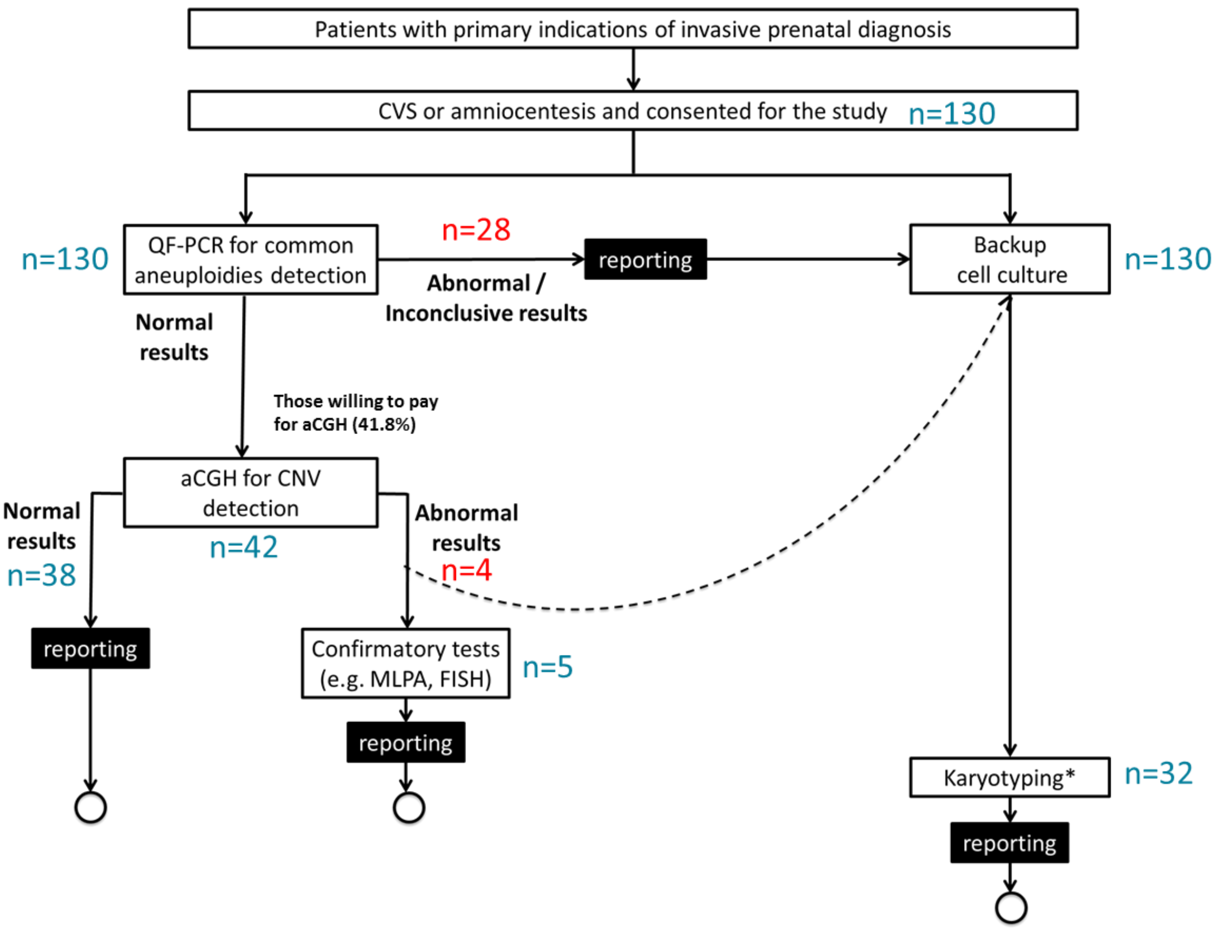

Supplementary figure 2b. Secondary analysis: detailed workflow of the current algorithm

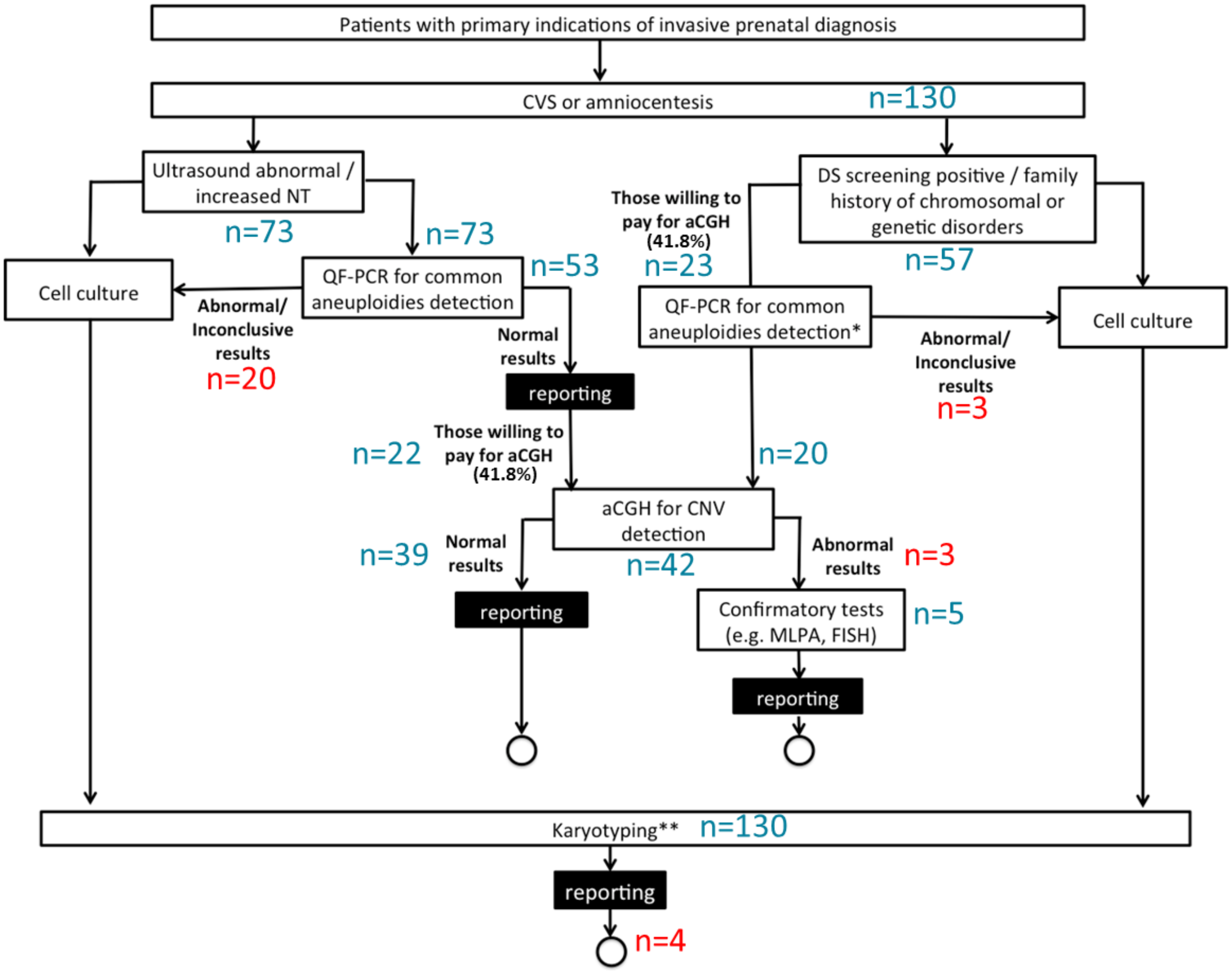

Supplement: Supplementary file 2 — Additional file 2: Figure S2a Secondary analysis: detailed workflow of the proposed algorithm. *Samples with inconclusive QF-PCR results and subsequent normal karyotyping results will proceed to aCGH on cultured cells. aCGH: array comparative genomic hybridization; CNV: copy number variation; CVS: chorionic villous sampling; FISH: fluorescence in-situ hybridization; MLPA: multiplex-ligation dependent probe amplification; QF-PCR: quantitative fluorescent polymerase chain reaction. Figure S2b Secondary analysis: detailed workflow of the current algorithm. *QF-PCR is not commonly offered free of charge for patients with primary indication of DS screening positive / family history of chromosomal or genetic disorders. However, for patients who are willing to pay for self-financed aCGH, the laboratory will first perform QF-PCR for common aneuploidies detection. If QF-PCR results abnormal, aCGH will not be proceeded.** Samples with inconclusive QFPCR results and subsequent normal karyotyping results will proceed to aCGH if patient is willing to pay for self-financed aCGH. aCGH: array comparative genomic hybridization; CNV: copy number variation; CVS: chorionic villous sampling; DS: Down syndrome; FISH: fluorescence in-situ hybridization; MLPA: multiplex-ligation dependent probe amplification; NT: nuchal translucency; QF-PCR: quantitative fluorescent polymerase chain reaction. [file 12884_2020_2772_MOESM2_ESM.pdf]
