# Supplementary material for: Cost-effectiveness analysis of chromosomal microarray as a primary test for prenatal diagnosis in Hong Kong
Source: BMC Pregnancy Childbirth. 2020 Feb 14;20:109. doi: 10.1186/s12884-020-2772-y (PMC7023733; doi:10.1186/s12884-020-2772-y)
Supplement: Supplementary file 3 — Additional file 3: Table S1 Primary indication of invasive testing for 130 prenatal cases. Table S2 Abnormal aCGH results and outcome (n = 11). [file 12884_2020_2772_MOESM3_ESM.pdf]

Supplementary table 1. Primary indication of invasive testing for 130 prenatal cases

	**Chorionic villi**		**Amniotic fluid**		**Total**	
**DS screening positive only**		32		21		53
**Fetal ultrasound abnormality**		16		35		51
Number of fetal anomalies						
One	4		21		25	
Two	6		8		14	
Three	2		3		5	
More than three	4		3		7	
**Increased NT >= 3.5 mm or cystic hygroma**		21		1		22
**Family history of chromosomal abnormality**		2		-		2
**Others**		2[a]		-		2
**Total**		73		57		130

[a]One non-invasive prenatal test (NIPT) positive; dichorionic diamniotic twin 1 with scan abnormality and twin 2 (this twin) normal

DS: Down syndrome; NT: nuchal translucency

Supplementary table 2. Abnormal aCGH results and outcome (n=11)

Case number	Gestation	Sample type	aCGH result (ISCN 2016)	Referring indication	Karyotype result	CNV size and type/classification, gene(s) or syndrome	Outcome / remarks
1	13+1	uncultured CV	arr[GRCh37] 13q31.2q34(89022234_115083595)x2~3	1st trimester DS screening risk 1:2; USS: Absent nasal bone, ? AVSD, exomphalos, bilateral talipes	47,XX,+mar dn[4]/46,XX[46]	• 26.06 Mb mosaic copy gain in 13q31.2-q34 / Pathogenic, mosaic partial trisomy 13q	TOP. No postmortem.
2	13+4	uncultured CV	arr[GRCh37] 21q22.11(34169002_35051732)x3 dn,21q22.3(45066431_48091216)x1 dn	1st trimester DS screening risk 1 in 210.	46,XY,r(21)(p11.2q22.3)dn	• 882.73kb copy gain in 21q22.11 / uncertain clinical significance<br>• 3.02Mb copy loss in 21q22.3 / Pathogenic, ring chromosome 21	Post test counselling by clinical geneticist. Livebirth. OFC <3rd at 13 months.
3	13+6	uncultured CV	arr[GRCh37] 18p11.32q23(146484_78013620)x2~3,22q11.1q13.33(17528442_51178150)x3	1st trimester DS screening risk 1 in 11, low PAPP-A 0.09 MoM	47,XX,+22	• Mosaic 77.87 Mb copy gain in 18p11.32-q23 / Pathogenic mosaic trisomy 18<br>• 33.65 Mb copy gain in 22q11.1-q13.33 / Pathogenic, trisomy 22	TOP. Abortus: no gross abnormality.
4	17+5	uncultured AF<br>cultured AF	arr[GRCh37] 8p23.3p12(202133_29191477)x1<br>arr[GRCh37] 8p23.3p12(202133_29191477)x1~2	USS: Oligohydramnios, LV < RV, LVOT not well seen, pericardial effusion, echogenic bowel, increased nuchal translucency (6mm at 11 weeks). NIPT: reduction in the amount of DNA from chromosome 8p.	46,XY,del(8)(p12)dn[16]/46,XY[15]	• 28.99Mb copy loss in 8p23.3-p12 (mosaic loss in cultured cells) / Pathogenic, terminal 8p deletion	TOP. No postmortem.
5	20+5	uncultured AF	arr[GRCh37] 1q43q44(239841348_249208146)x3,4q34.3q35.2(182344275_190896675)x1	USS: Fetal diaphragmatic hernia	46,XY.ish der(4)(D4Z1+,D4S2930–,VIJyRM2123+)	• 9.37 Mb copy gain in 1q43-q44<br>• 8.55 Mb copy loss in 4q34.3-q35.2/<br>Pathogenic unbalanced translocation<br>(metaphase FISH confirmed the unbalanced translocation.	Decided to keep pregnancy. Joint counselling with paediatric surgeon. Defaulted follow up since 25 weeks. Declined parental karyotype.

6	19+3	uncultured AF	arr[GRCh37] Xp21.2p21.1(31464899_31522762)x0 mat	1st trimester DS screening risk 1:230.	46,XY	• 57.86 kb deletion in Xp21.2-p21.1, involving *DMD* gene / Pathogenic, Dystrophinopathy	Post-test counselling with neurologist. Livebirth. BMD. Borderline global delay at 19 months.
7	23+4	uncultured AF	arr[GRCh37] 16p13.11(15125829_16287900)x3 pat	USS: Fetal intracranial tumor	46,XX	• 1.16 Mb copy gain in 16p13.11 / Pathogenic, 16p13.11 duplication syndrome	TOP. No postmortem. Post-TOP counselling by clinical geneticist. Normal paternal phenotype.
8	13+4	uncultured CV	arr[GRCh37] 16p11.2(29657192_30188269)x1 dn	1st trimester DS screening risk 1:90	46,XX	• 531.08 kb copy loss in 16p11.2 / Pathogenic, 16p11.2 microdeletion syndrome	Post-test counselling by clinical geneticist. TOP. No postmortem.
9	20	uncultured AF	arr[GRCh37] 16p11.2(29657192_30188269)x1 dn	NIPT low risk. USS: multiple level hemivertebra, 1A1V, right ventricle hypoplasia with tricuspid atresia.	46,XX	• 531.08 kb copy loss in 16p11.2 / Pathogenic, 16p11.2 microdeletion syndrome	TOP. Postmortem: right ventricular hypoplasia, tricuspid atresia, ASD, hemivertebrae. Maternal aCGH showed mosaic 9p13.2 deletion confirmed on FISH. Post TOP counselling by clinical geneticist. Normal maternal phenotype.
10	22+4	uncultured AF	arr[GRCh37] 16p11.2(29657192_30188269)x1 dn	USS: TOF, possibility of transposition of great artery.	46,XY	• 531.08 kb copy loss in 16p11.2 / Pathogenic, 16p11.2 microdeletion syndrome	TOP. Postmortem: Tetralogy of Fallot, right sided aortic arch, atretic ductus arteriosus, cerebellar hypoplasia.
11	20+1	uncultured AF	arr[GRCh37] 2q34(212660463_213736135)x3 pat	NIPT low risk. USS: spine abnormality, Right club foot.	46,XX	• 1.08 Mb copy gain in 2q34 / Uncertain clinical significance	TOP. No postmortem. Babygram: hemivertebra, non-segmentation defect, 11 pairs of ribs, bilateral clubfeet. Normal paternal phenotype.

aCGH: array comparative genomic hybridization; AF: amniotic fluid; AVSD: atrioventricular septal defect; BMD: Becker muscular dystrophy; CNV: copy number variant; CV: chorionic villi; DS: Down syndrome; BMD: Becker muscular dystrophy; FISH: fluorescence in-situ hybridization; MoM: multiples of median; NIPT: non-invasive prenatal testing; PAPP-A pregnancy associated plasma protein-A; TOF: Tetralogy of Fallot; TOP: termination of pregnancy; USS: ultrasound scan
